# Reason for hospitalization contrasting adjudication versus ICD-10-CM coding among persons with HIV, 2016–2019

**DOI:** 10.1186/s12981-026-00855-8

**Published:** 2026-02-18

**Authors:** Alexander C. Commanday, Lindsay E. Browne, Amanda E. Moy, Heather I. Henderson, John P. Franzone, Geargin B. Wilson, Claire E. Farel, Darren A. Dewalt, Joseph J. Eron, Sonia Napravnik

**Affiliations:** 1 Department of Medicine, School of Medicine, University of North Carolina at Chapel Hill, 130 Mason Farm Road, Bioinformatics Building CB#7030, Chapel Hill, NC 27599-7030 USA; 2 Department of Epidemiology, Gillings School of Global Public Health, University of North Carolina at Chapel Hill, 130 Mason Farm Road, Bioinformatics Building CB#7030, Chapel Hill, NC 27599-7030 USA

**Keywords:** HIV, AIDS, Hospitalization, Reason for hospitalization, ICD-10, Principal diagnosis

## Abstract

**Background:**

People with HIV (PWH) have disproportionately high hospitalization rates, the reasons for which are incompletely understood. Hospitalization reasons based on International Classification of Diseases, Tenth Revision, Clinical Modification (ICD-10-CM) discharge codes may lead to misclassification. We evaluated the performance of ICD-based reason for hospitalization with adjudication in PWH.

**Methods:**

We randomly sampled 302 of 1428 hospitalizations (21%) between 2016 and 2019 among PWH in the UNC CFAR Clinical Cohort Study (UCHCC). ICD-based reason was defined as first-listed ICD discharge diagnosis, or second if the principal diagnosis was HIV. We contrasted ICD-based reason and adjudication with four outcomes: complete agreement (same category and diagnosis), partial agreement (same category, different diagnosis), disagreement (different category and diagnosis), and inadequate ICD coding (no single ICD-10-CM code was appropriate).

**Results:**

Overall the 302 hospitalizations occurred among 204 PWH, 69% of whom were male, with median age 51 years (interquartile range [IQR], 40–58), and CD4 count 539 (IQR, 242–789), at time of hospitalization. Adjudication completely agreed with ICD-based reason in 73% hospitalizations, partially agreed in 14%, disagreed in 8%, and ICD codes were inadequate in 6%. Patient characteristics associated with lower proportion of complete or partial agreement were recent CD4 count < 200, nadir CD4 < 50, ≥ 15 discharge diagnoses, length of stay ≥ 4 days, and HIV as a principal diagnosis.

**Conclusions:**

Overall ICD codes performed well in capturing reason for hospitalization in most cases. However, adjudications are needed among certain groups of patients, including those with poorly controlled HIV and complex hospitalization course.

**Supplementary Information:**

The online version contains supplementary material available at 10.1186/s12981-026-00855-8.

## Introduction

The introduction of effective combination anti-retroviral therapy (ART) in the late 1990s marked a turning point in the care of people with HIV (PWH), dramatically improving life expectancy to near parity with the general population [1, 2]. Alongside these gains, hospitalization rates have declined, driven largely by reductions in Acquired Immunodeficiency Syndrome (AIDS)-defining illnesses (ADIs) [3–12]. Yet, despite these advances, PWH continue to experience higher hospitalization rates than the general population—comparable to those seen among older individuals [2, 8, 11, 13]. This persistent disparity is likely influenced by a growing burden of comorbidities, age-related conditions, and health inequities. However, the contemporary drivers of hospitalization among PWH remain incompletely understood [2, 14, 15]. Prior studies, relying on International Classification of Disease (ICD) discharge diagnosis codes, grouped by category or organ system, indicate that non-AIDS-defining infections, followed by cardiovascular and gastrointestinal illness, are the leading causes of hospitalization in PWH in the US [3–5].

ICD-10-CM codes (ICD, Tenth Revision, Clinical Modification, hereafter referred to as ICD codes) were widely adopted in United States hospitals in late 2015 and are utilized for multiple purposes including hospital coding, billing, institutional quality improvement and reporting, and research [16–18]. While ICD codes offer a standardized approach to medical record summarization, their accuracy in identifying the primary reason for hospitalization in PWH compared to the reference standard method of medical record adjudication is not well understood.

In this study, we evaluated the accuracy of ICD codes for determining reason for hospitalization, compared with medical record adjudication, in a sample of hospitalizations among PWH in a clinical cohort in the Southeastern United States from 2016 to 2019.

## Methods

### Study population

This study was based in the University of North Carolina (UNC) Center for AIDS Research HIV Clinical Cohort (UCHCC), which prospectively collects electronic health record (EHR) data including demographic information, laboratory measures, medications, and adjudicated clinical comorbidities, as well as hospitalization information from 10 UNC-affiliated hospitals with a large catchment area across North Carolina [4]. Patients provide written informed consent to participate in the UCHCC, and this study was approved by the UNC Institutional Review Board.

Hospital admissions were included if they spanned at least one midnight and were assigned at least one ICD discharge diagnosis code. Hospitalizations were excluded if they were related to childbirth, did not have a discharge summary available, or if the participant was transferred to another hospital prior to discharge. The study period was chosen to start with the introduction of ICD-10-CM and exclude the COVID-19 pandemic period. A random sample of 20% of all hospital admissions occurring during the study period was selected for adjudication.

### Study measures

Demographic and clinical factors were collected using available data elements in the UCHCC. All factors were measured at time of admission, including age, race/ethnicity, sex, clinical comorbidities, recent HIV clinic visit, adjudicated ART regimens, and laboratory measures including most recent HIV RNA level (defined as detectable if > 50 copies/mL), most recent CD4 cell count within 12 months of the index hospitalization, and nadir CD4 cell count. Hospitalization factors included discharge diagnosis ICD codes, admission diagnosis ICD code (assigned by the admitting service during hospitalization), number of discharge diagnoses, length of stay, and assigned admission-discharge-transfer (ADT) hospitalization class (inpatient, observation, inpatient psych, or extended recovery). Length of stay was calculated as discharge date minus admission date plus 1.

We created a hospitalization medical record adjudication protocol where adjudication determined the reason for admission, defined by the Uniform Hospital Discharge Data Set (UHDDS) as “that condition established after study to be chiefly responsible for occasioning the admission of the patient to the hospital for care”, and assigned the most appropriate ICD code, if available, not including HIV disease (‘B20’) [18, 19]. The diagnosis was further categorized by organ system based on modified Clinical Classification Software (CCS) as previously described, which recategorizes AIDS-defining illnesses and non-AIDS-defining infections [3, 5, 20]. The adjudicator could specify that a single ICD code was inadequate to characterize reason for hospitalization and provide one or more rationales. Hospitalizations were flagged for discussion by the primary adjudicator for further review by a panel of 3–4 HIV physicians to achieve a consensus.

ICD-based reason for hospitalization was defined as the first-ranked ICD discharge diagnosis; if this was HIV disease (‘B20’) or asymptomatic HIV infection status (‘Z21’) the second listed ICD code was chosen. These diagnoses were further categorized by organ system, with modifications detailed above [3, 5]. Adjudicated reason for hospitalization and diagnosis category were compared to the ICD-based reason with four possible outcomes: complete agreement of ICD code and diagnosis category, partial agreement (different ICD codes but same diagnosis category), disagreement (different ICD codes and diagnosis categories), and ICD codes inadequate to capture the reason for hospitalization. Results were further dichotomized into agreement (complete and partial agreement) and no agreement (disagreement and ICD codes inadequate).

### Statistical analysis

Proportion agreement of ICD-based reason for hospitalization and adjudicated reason for hospitalization was calculated as number of agreements divided by total number of hospitalizations adjudicated. We also calculated sensitivity and estimated differences in the proportion agreement stratified by patient and hospitalization characteristics and estimated 95% confidence intervals (CI) as measures of precision. The Chi-square test was used to test for differences in proportions. Analyses were conducted in Stata 18 (StataCorp LLC, College Station, TX).

## Results

### Hospitalization characteristics

Between 2016 and 2019, a total of 1,428 hospitalizations among 599 unique UCHCC participants met study inclusion criteria. A random sample of 321 (22.5%) hospitalizations were selected for adjudication; 14 hospitalizations were excluded for having no available discharge summary and 5 for being related to childbirth. The 302 remaining hospitalizations were among 204 unique patients, with 141, 39, and 24 patients contributing 1, 2, and 3 or more hospitalizations, respectively. At time of each hospital admission, the median age was 51 years (interquartile range [IQR], 40–58) and 208 (68.9%) of admissions were among men (Table 1). The median recent CD4 cell count was 539 cells/mm^3^ (IQR, 242–789), median nadir CD4 cell count was 129 (IQR, 30–323), and 82 (27.2%) patients had a detectable viral load at admission. Over two-thirds (67.2%) of hospitalizations occurred at UNC Medical Center, with the remainder occurring at UNC-affiliated hospitals. A majority were assigned ‘inpatient’ ADT class (70.2%), the median length of stay was 4 days (IQR, 3–7), and median number of discharge diagnoses was 15 (IQR, 10–20).

**Table 1:** Patient demographics and clinical characteristics at time of hospital admission, among 302 hospitalizations

	N (%)
Age	
Under 55	190 (62.9%)
55 and over	112 (37.1%)
Sex	
Female	94 (31.1%)
Male	208 (68.9%)
Preadmission CD4^a^	
200 or greater	207 (79.0%)
Less than 200	55 (21.0%)
Nadir CD4	
50 or greater	206 (68.2%)
Less than 50	96 (31.8%)
Last viral load detectable^b^	
No	220 (72.8%)
Yes	82 (27.2%)
Last HIV clinic visit within 12 months	
No	54 (17.9%)
Yes	248 (82.1%)
Hospitalization class^c^	
Inpatient	212 (70.2%)
Observation	53 (17.5%)
Inpatient psych	18 (6.0%)
Extended recovery	19 (6.3%)
Hospital length of stay	
< 4 days	107 (35.4%)
4 to 7 days	124 (41.1%)
> 7 days	71 (23.5%)
Number of discharge diagnoses	
< 15	146 (48.3%)
≥ 15	156 (51.7%)
Top diagnosis categories by ICD-based method^d^	
Infection	67 (22.2%)
Cardiovascular	43 (14.2%)
Musculoskeletal	24 (7.9%)
Neoplasms	21 (7.0%)
Psychiatric	21 (7.0%)
Gastrointestinal	20 (6.6%)

^a^Included if obtained within 365 days prior to admission. CD4 measured in cells/mm^3^

^b^Detectable if > 50 copies/mL

^c^Assigned Admission-Transfer-Discharge (ADT) hospitalization class

^d^Diagnosis category determined by modified Clinical Classifications Software applied to ICD-based reason for hospitalization, with reorganized infections and AIDS-defining illnesses, top diagnosis categories shown

### Adjudication outcomes

**Fig. 1:**
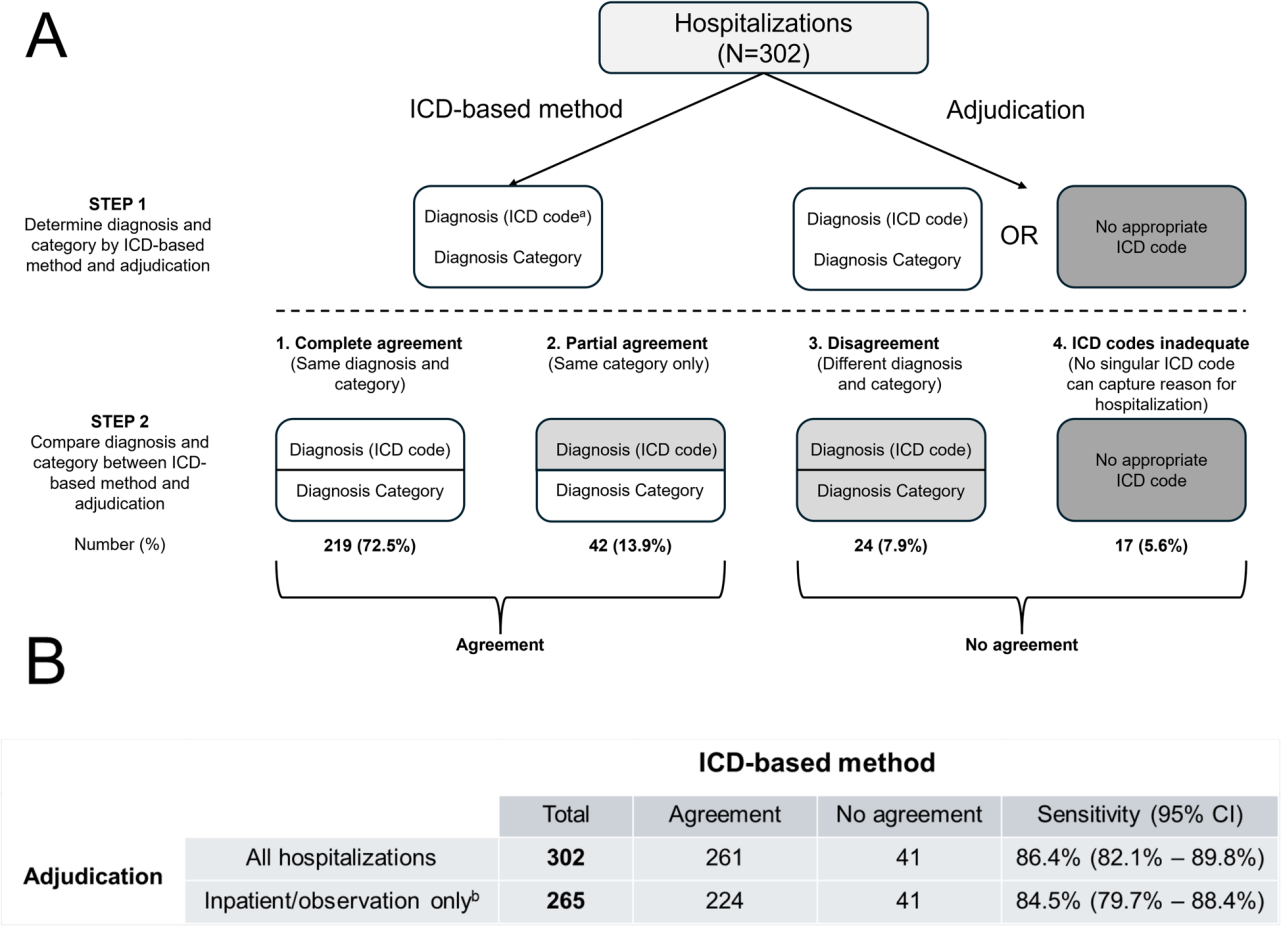
Flowchart for comparing adjudication and ICD-based reason for hospitalization. **A** Four possible outcomes contrasting adjudication and ICD-based methods in determining reason and category of hospitalization. Numbers and percentages are shown for each of the four outcomes. **B** Dichotomized outcomes by agreement are shown, with calculated sensitivity and 95% confidence intervals. ^a^International Classification of Disease, 10th revision, Clinical Modification. ^b^Excluding 37 hospitalizations with Admission/Discharge/Transfer class ‘inpatient psych’ and ‘extended recovery’

When comparing adjudication and the ICD-based method, the reason for hospitalization agreed completely in 219 of 302 cases (72.5%), partially in 42 cases (13.9%), and disagreed in 24 cases (7.9%) (Fig. 1A). Specific findings among the cases with disagreement are presented in Supplemental Table. Based on adjudications ICD codes were inadequate to capture the reason for hospitalization in 17/302 (5.6%) of hospitalizations; the most common rationales were multiple overlapping qualifying conditions of different diagnosis categories [8], unusual presentations not captured in ICD-10 codes [4], diagnostic uncertainty [4], and multiple minor contributing factors [3], with more than one reason recorded for some hospitalizations. The 37 hospitalizations with assigned ADT classes of ‘inpatient psych’ and ‘extended recovery’ yielded 100% agreement (complete or partial) of adjudication and ICD-based method; thus, these were excluded from further analyses. Among the 265 remaining hospitalizations, we observed complete agreement, partial agreement, disagreement, and inadequate ICD codes among 69.4%, 15.1%, 9.1%, and 6.4% respectively. After combining complete or partial agreement groups and the groups where the ICD-based method either disagreed with adjudication or ICD codes were inadequate, we observed a sensitivity of 84.5% (95% CI, 79.7% to 88.4%) (Fig. 1B).

**Fig. 2:**
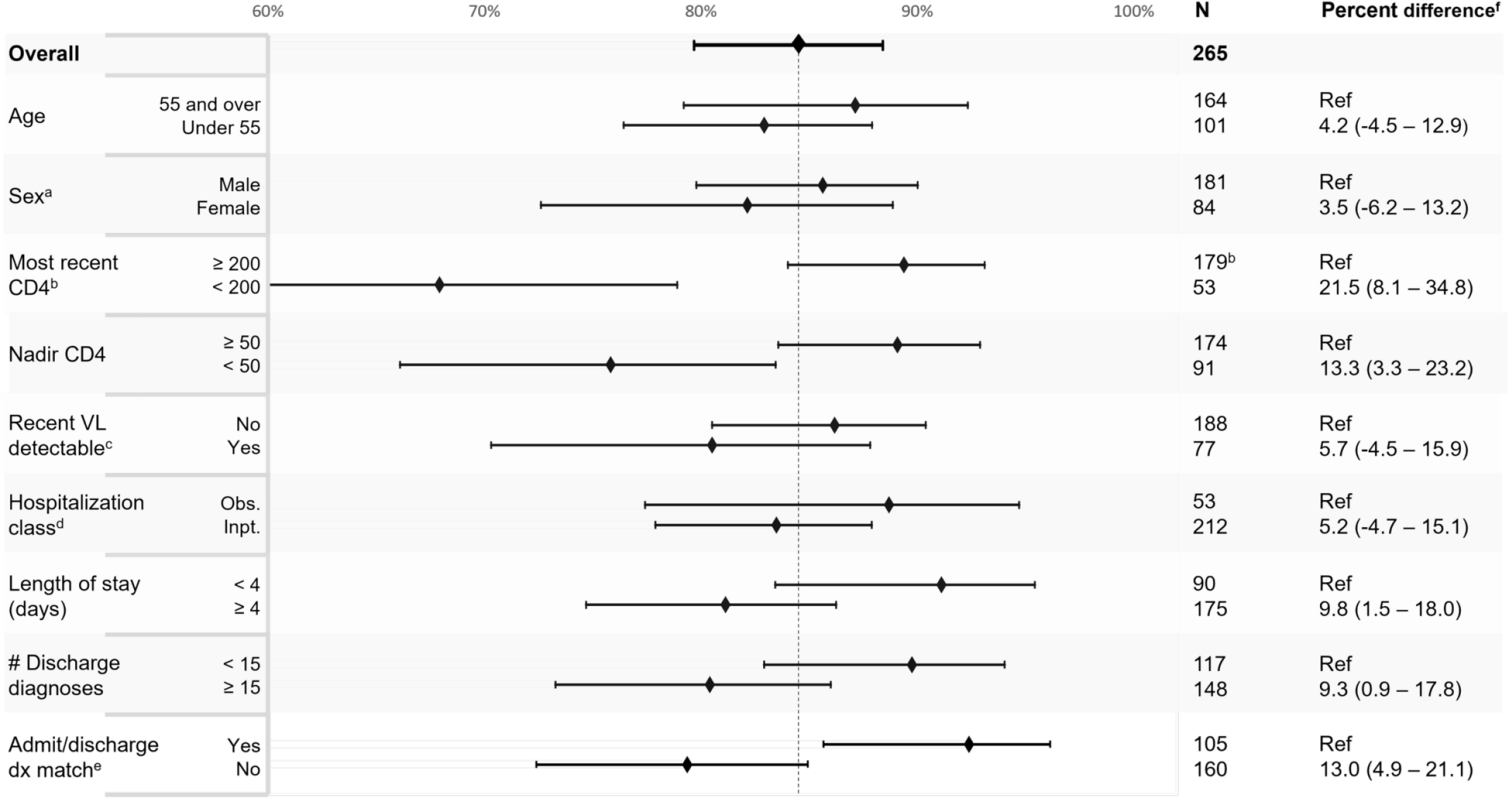
Percent agreement between adjudication and ICD-based reason for hospitalization, stratified by patient and hospitalization characteristics. Percentage of complete or partial agreement versus disagreement or inadequate ICD codes comparing adjudication and ICD-based reason, stratified by patient and hospitalization characteristics. ^a^Sex. ^b^Measured in cells/mm^3^. Included if within 365 days prior to admission. ^c^Detectable if > 50 copies/mL. ^d^Assigned Admission/Transfer/Discharge class, inpatient psych and extended recovery excluded from analysis, observation and inpatient included. ^e^Matching admission and first listed discharge diagnosis. ^f^Percent difference in agreement compared to referent category, with 95% confidence intervals. VL = viral load; dx = diagnosis; ICD = international classification of disease

Hospitalizations with recent CD4 count < 200 cells/mm^3^, nadir CD4 cell count < 50 cells/mm^3^, length of stay 4 days or greater, 15 or more discharge diagnoses, and non-matching admission and discharge diagnoses were associated with lower proportion of agreement (Fig. 2). The greatest differences in agreement were for recent (≥ vs < 200) and nadir (≥ vs < 50) CD4 cell counts with 21.5% agreement difference (95% CI, 8.1% to 34.8%) and 13.3% (95% CI, 3.3% to 23.2%), respectively. Additional characteristics associated with at least a 5% difference in agreement were detectable VL and inpatient ADT class, although these contrasts were not statistically significant. We also compared hospitalizations where HIV disease was the first-listed discharge diagnosis, since in our analyses we used the second-listed discharge diagnosis in these cases. Among these 24 hospitalizations we observed 58% agreement with adjudication and a percent difference of 29% (95% CI, 9% to 49%) compared to those without HIV disease as the first-listed discharge diagnosis.

### Reasons for hospitalization

Using the ICD-based method, the top reasons for hospitalization were non-ADI infections and cardiovascular conditions (Supplemental Figure). Musculoskeletal, psychiatric, neoplasms, GI/liver, respiratory and GU/renal conditions each also accounted for at least 5% of all hospitalizations. Heterogeneity in agreement was observed across diagnosis categories, with substantial disagreement for ADIs and non-ADI infections, although limited sample sizes precluded statistical analyses. The 3 leading ICD discharge diagnosis codes by the ICD-based method were: ‘Sepsis, unspecified organism’ (N = 14), ‘Chronic obstructive pulmonary disease with acute exacerbation’ (N = 10), and ‘Encounter for antineoplastic chemotherapy’ (N = 8).

## Discussion

In this retrospective cohort study of 302 hospitalizations among 204 PWH from 2016 to 2019, the reason for hospitalization established by medical record adjudication agreed with the ICD-based reason in 86.4% of admissions. Characteristics associated with lower agreement of the ICD-based method were related to complex and HIV-associated hospitalizations, which may require adjudication for accurate estimation of reason for hospitalization.

Studies of hospitalization reasons among PWH have relied almost exclusively on ICD discharge codes [3–12]. To the best of our knowledge one study compared ICD codes to adjudications for assigning hospitalization reasons, but was performed from 1995 to 1998, when opportunistic infections accounted for an overwhelming proportion of hospitalizations [6]. ICD codes have also been used for assessing reasons for hospitalization in other patient cohorts, including in patients with rheumatologic diseases [21–25] or hospital readmission after stroke [26]; however, they have not been compared to adjudication. ICD codes and algorithms incorporating ICD codes and other clinical data have been validated for specific clinical conditions using adjudication (e.g. myocardial infarction, heart failure, hemorrhagic stroke, pulmonary embolism, pneumonia, and sepsis) [27–36]. These studies are limited in their scope for each specific condition and do not focus solely on the principal diagnosis.

Patients with poorly-controlled, advanced, or recently diagnosed HIV are at increased risk of hospitalization [3–9, 37]. Our study found that hospitalizations of patients with preadmission CD4 counts below 200 cells/mm^3^ had lower agreement of the ICD-based method in determining reasons for hospitalization. There are multiple potential reasons for the worse performance of the ICD-based method among participants with advanced HIV, including inconsistent coding strategies and increased hospitalization complexity. The ICD-based method utilizes the second listed diagnosis code for primary reason for hospitalization if the principal diagnosis is HIV disease [4, 5]. ICD-10-CM guidelines from the Centers for Medicare and Medicaid Services (CMS)[18] recommend using HIV as a principal diagnosis with admissions for HIV-associated conditions, followed by the associated condition. However we observed that using the second listed ICD in these situations leads to very poor agreement with adjudication (58.3%). Our protocol allowed an adjudicator to specify that ICD codes were inadequate to determine the reason for hospitalization, most commonly attributing this selection to multiple overlapping qualifying conditions of different diagnosis categories. By virtue of advanced immunosuppression, hospitalizations of patients with uncontrolled HIV may be characterized by multiple concomitant AIDS-defining illnesses and complications not reflected well in ICD-10-CM codes.

The top diagnosis categories we identified were comparable to those from other studies, although additional data is needed to evaluate specific trends with regards to ICD-based method performance [3–5, 7–10]. The most common primary diagnosis code in our study was ‘sepsis, organism unspecified’, in line with findings in other PWH cohorts [3], and among people without HIV [38].

Our adjudication process included protocolized review of EHR data to identify reason for hospitalization; however, a limitation of this work is there were no specific qualifying criteria for individual diagnosis codes. As expected, additional disagreement was observed when we defined agreement based on a specific ICD code, rather than a diagnosis category. Since physician-performed adjudications can introduce bias, we incorporated a discussion panel of HIV physicians to evaluate more complex hospitalizations. Other indicators of hospitalization complexity—such as comorbidities, ICU stays, transfers, consults, and procedures [39]—may help identify limitations in the ICD-based method, though these elements are often underrepresented in abstracted data and typically need adjudication for validation.

The study period was chosen to exclude the use of ICD-9 codes and the COVID-19 pandemic [18, 40]. Additional work including more recent hospitalizations is needed to characterize any changes, pre-, during and post- the COVID-19 pandemic, including changes in health care provision and funding. We were unable to assess post-hospitalization discharge planning, an area of important inquiry given the high burden of hospital readmissions in the United States. Our study was also conducted at one health care center, although covering a wide and diverse patient population and geographic area. Inclusion of additional health care centers, as well as individuals with a variety of health insurance coverage, and across different areas of the United States, is needed to confirm generalizability.

Expansion of this work to capture all hospitalizations a patient has experienced is also indicated. This is especially important to characterize patients with high hospitalization utilization where outpatient interventions may be especially needed. Additionally, an approach that can accurately identify hospitalizations that require adjudication, versus those where an ICD-based approach is sufficient, would enable targeted implementation of adjudications that is feasible on a larger scale.

## Conclusions

An ICD-based method may be appropriate for assessing reason for hospitalization of PWH in most cases. However, adjudication is needed for specific hospitalizations, especially those among people with poorly controlled HIV and complex hospitalization courses. Our findings underscore the importance of integrating both structured data and expert review in studies aiming to characterize hospitalization patterns among PWH.

## Supplementary Information

Supplementary Material 1.

Supplementary Material 2.

## Data Availability

The datasets used and analyzed during the current study are available from the corresponding author on reasonable request.

## Abbreviations

HIV: Human immunodeficiency virus
ART: Anti-retroviral therapy
PWH: People with HIV
AIDS: Acqiured immunodeficiency syndrome
CFAR: Center for AIDS research
ADI: AIDS-defining illness
ICD-10-CM: International classification of disease 10th revision, clinical modification
ADT: Admission-discharge-transfer
IQR: Interquartile range
EHR: Electronic health record
UNC: University of North Carolina
UCHCC: University of North Carolina, Center for AIDS Research, Clinical Cohort Study
UHDDS: Uniform Hospital Discharge Data Set
CCS: Clinical Classification Software
CI: Confidence interval
CMS: Centers for Medicare and Medicaid Services
