# Supplementary material for: Reason for hospitalization contrasting adjudication versus ICD-10-CM coding among persons with HIV, 2016–2019
Source: AIDS Res Ther. 2026 Feb 18;23:39. doi: 10.1186/s12981-026-00855-8 (PMC13020228; doi:10.1186/s12981-026-00855-8)
Supplement: Supplementary file 1 — Supplementary Material 1. [file 12981_2026_855_MOESM1_ESM.pptx]

## Slide 1
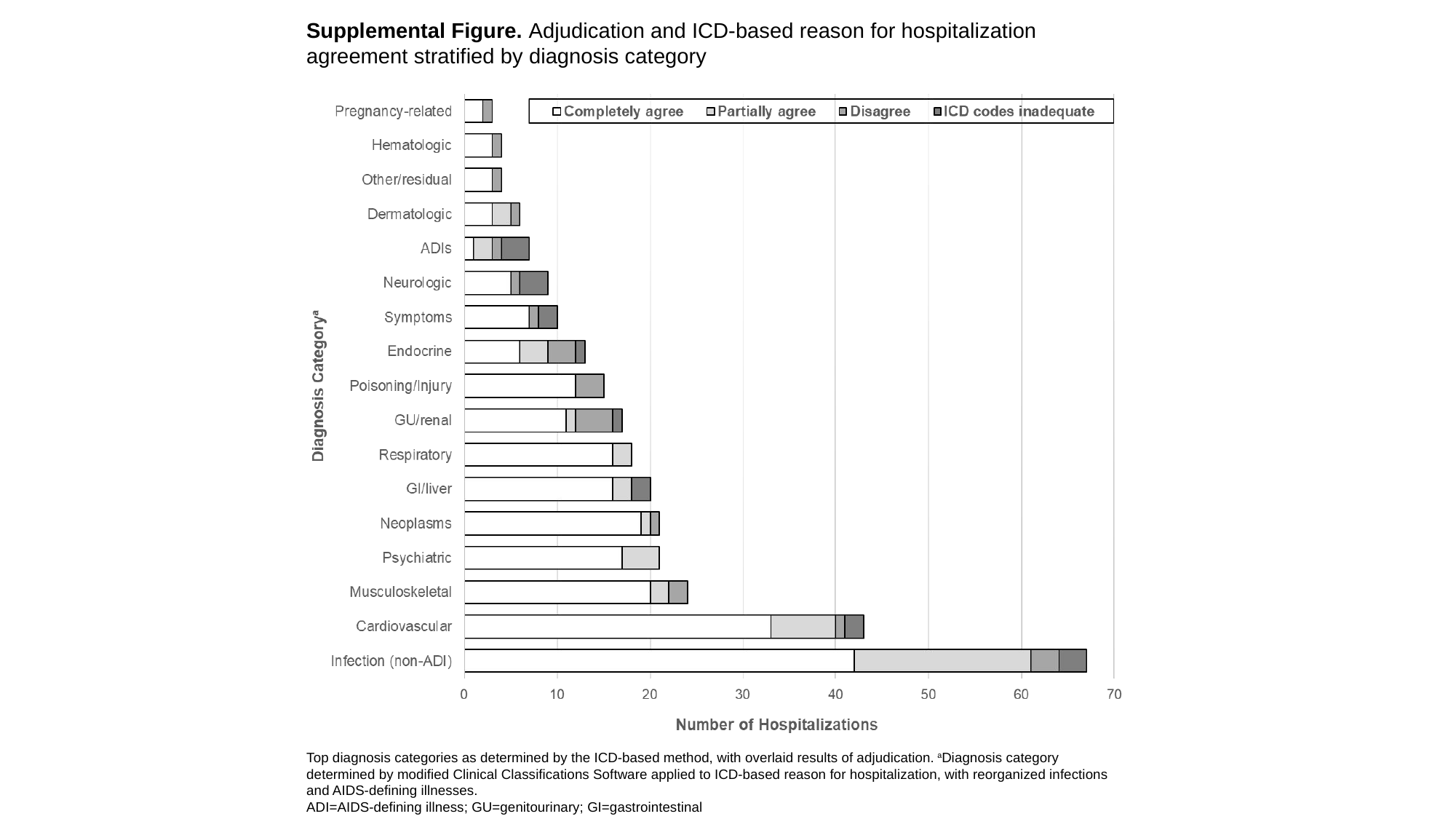

Supplemental Figure. Adjudication and ICD-based reason for hospitalization agreement stratified by diagnosis category
Top diagnosis categories as determined by the ICD-based method, with overlaid results of adjudication. aDiagnosis category determined by modified Clinical Classifications Software applied to ICD-based reason for hospitalization, with reorganized infections and AIDS-defining illnesses.
ADI=AIDS-defining illness; GU=genitourinary; GI=gastrointestinal
