# Supplementary material for: Reason for hospitalization contrasting adjudication versus ICD-10-CM coding among persons with HIV, 2016–2019
Source: AIDS Res Ther. 2026 Feb 18;23:39. doi: 10.1186/s12981-026-00855-8 (PMC13020228; doi:10.1186/s12981-026-00855-8)
Supplement: Supplementary file 2 — Supplementary Material 2. [file 12981_2026_855_MOESM2_ESM.pdf]

Supplemental Table: ICD codes and categories of N=24 hospitalizations with disagreement between ICD-based method and adjudication

ICD-based reason	ICD-based category	Adjudicated reason	Adjudicated category
Tachypnea, not elsewhere classified (R06.82)	Symptoms	Acute and chronic respiratory failure with hypoxia (J96.21)	Respiratory
Dental caries, unspecified (K02.9)	Other/residual	Hypertensive urgency (I16.0)	Cardiovascular
Diseases of the digestive system complicating the puerperium (O99.63)	Pregnancy-related	Unspecified acute appendicitis (K35.80)	Gastrointestinal/liver
Hypertensive chronic kidney disease with stage 5 chronic kidney disease or end stage renal disease (I12.0)	Genitourinary/renal	Respiratory failure, unspecified, unspecified whether with hypoxia or hypercapnia (J96.90)	Respiratory
Pressure ulcer of sacral region, stage 4 (L89.154)	Dermatologic	Methicillin susceptible staphylococcus aureus infection, unspecified site (A49.01)	Non-AIDS-defining infection
Malignant neoplasm of unspecified part of right bronchus or lung (C34.91)	Neoplasms	Lymphoid interstitial pneumonia (J84.2)	Respiratory
Bursitis of right shoulder (M75.51)	Musculoskeletal	Other infective bursitis, shoulder (M71.11)	Non-AIDS-defining infection
End stage renal disease (N18.6)	Genitourinary/renal	Syncope and collapse (R55)	Symptoms
Sepsis, unspecified organism (A41.9)	Non-AIDS-defining infection	Noninfective gastroenteritis and colitis, unspecified (K52.9)	Gastrointestinal/liver
Type 2 diabetes mellitus with diabetic chronic kidney disease (E11.22)	Endocrine/nutritional	Acute kidney failure, unspecified (N17.9)	Genitourinary/renal
Sarcoidosis of other sites (D86.89)	Musculoskeletal	Disseminated mycobacterium avium-intracellulare complex (DMAC) (A31.2)	AIDS-defining illness
Sepsis, unspecified organism (A41.9)	Non-AIDS-defining infection	Kaposi's sarcoma of lung (C46.5)	AIDS-defining illness
Other specified complication of other internal prosthetic devices, implants and grafts, initial encounter (T85.898A)	Poisoning/injury	Sepsis, unspecified organism (A41.9)	Non-AIDS-defining infection

Candidal esophagitis (B37.81)	AIDS-defining illness	Urinary tract infection, site not specified (N39.0)	Non-AIDS-defining infection
Tubulo-interstitial nephritis, not specified as acute or chronic (N12)	Genitourinary/renal	Polycystic kidney, adult type (Q61.2)	Congenital
Type 2 diabetes mellitus with other specified complication (E11.69)	Endocrine/nutritional	Other osteomyelitis, other site (M86.8X8)	Non-AIDS-defining infection
Poisoning by cocaine, accidental (unintentional), initial encounter (T40.5X1A)	Poisoning/injury	Other chest pain (R07.89)	Cardiovascular
Hemiplegia, unspecified affecting left nondominant side (G81.94)	Neurologic	Cerebral infarction due to thrombosis of right middle cerebral artery (I63.311)	Cardiovascular
Type 1 diabetes mellitus with other skin complications (E10.628)	Endocrine/nutritional	Cutaneous abscess of left lower limb (L02.416)	Non-AIDS-defining infection
Pneumonia, unspecified organism (J18.9)	Non-AIDS-defining infection	Acute and chronic respiratory failure with hypoxia (J96.21)	Respiratory
Hypertensive heart and chronic kidney disease with heart failure and stage 1 through stage 4 chronic kidney disease, or unspecified chronic kidney disease (I13.0)	Cardiovascular	Acute on chronic diastolic (congestive) heart failure (I13.0)	Cardiovascular
Inflammatory conditions of jaws (M27.2)	Musculoskeletal	Periapical abscess without sinus (K04.7)	Non-AIDS-defining infection
Contusion of right thigh, initial encounter (S70.11XA)	Poisoning/injury	Nontraumatic soft tissue hematoma (M79.81)	Musculoskeletal
Hypertensive heart and chronic kidney disease with heart failure and with stage 5 chronic kidney disease, or end stage renal disease (I13.2)	Cardiovascular	Pneumonia, unspecified organism (J18.9)	Non-AIDS-defining infection
